# Evaluations of Interventions with Child Domestic Workers: A Rapid Systematic Review

**DOI:** 10.3390/ijerph181910084

**Published:** 2021-09-25

**Authors:** Nambusi Kyegombe, Nicola S. Pocock, Clara W. Chan, Jonathan Blagbrough, Cathy Zimmerman

**Affiliations:** 1 Department of Global Health and Development, London School of Hygiene and Tropical Medicine, London WC1H 9SH, UK; nicola.pocock@lshtm.ac.uk (N.S.P.); cathy.zimmerman@lshtm.ac.uk (C.Z.); 2 Lumos Foundation, Peninsular House, London EC3R 8NB, UK; 3 Public Health England, Wellington House 133-155, Waterloo Road, London SE1 8UG, UK; clara.chan1@alumni.lshtm.ac.uk; 4 Children Unite, London E9 7JR, UK; jonathan.blagbrough@childrenunite.org.uk

**Keywords:** PRISMA, child domestic workers, interventions, girls’ and adolescents’ health, education, child protection, child labour, labour exploitation, domestic servitude, gender

## Abstract

Little is known about interventions to support the education, skills training, and health of female child domestic workers (CDWs). This rapid systematic literature review followed PRISMA guidelines (PROSPERO registration: CRD42019148702) and summarises peer-reviewed and grey literature on health, education, and economic interventions for CDWs and interventions targeting employers. We searched six electronic databases and purposively searched grey literature. We included observational studies, which included an intervention, quasi-experimental, and experimental studies. Two reviewers independently screened articles. Data were extracted on intervention description, inputs, activities, type of evaluation, outcomes, effect size or impact where applicable, limitations, and ethical considerations. All studies were quality appraised. We identified eight papers from five studies. Six papers reported on health-related outcomes, two on education-related outcomes, and three on economic outcomes. No evaluations of employer-related interventions were identified. Only one intervention specifically targeted CDWs. Others included CDWs in their sample but did not disaggregate data for CDWs. Findings suggest that the evaluated interventions had a limited impact on CDW’s health, education, and economic outcomes. While it appears feasible to reach CDWs with outreach interventions, further work is needed to improve the consistency of their effectiveness and their ability to improve CDWs’ current and future prospects.

## 1. Introduction

Internationally, ‘child domestic workers’ (CDW) have been broadly understood to be children and young people under the age of 18 who ‘work in other people’s households doing domestic chores, caring for children and running errands, among other tasks’ [1].

The most recent global estimates for CDW suggest that 17.2 million children (aged 5–17 years) are in paid or unpaid domestic work in households other than their own [2]. More than two-thirds of these children are girls. Domestic work is a highly gendered phenomenon, which is closely associated with unpaid female household reproductive labour at one end and the continuum between unpaid domestic labour and fairly paid adult work options for women outside the home at the other [3,4,5]. Furthermore, the latest global estimates for child labour indicate that 11.5 million CDWs are in situations of child labour, whether due to their age or the hazardous work they carry out [6].

Entry into child domestic work often begins when children and young people, especially girls, are relocated via formal and informal labour brokers or decide independently to move from their homes to work and ‘live-in’ with an employer, commonly from rural to urban areas. This decision can represent a household survival strategy, an attempt to pursue an education, a kinship relationship or obligation, or is based on parents’ beliefs that domestic work is a safe and suitable option for daughters [7,8,9]. Jones et al. (2018) indicate that restrictive gender norms, particularly for girls, are prominent drivers of girls’ migration [10]. For some girls from rural areas, moving to an urban area to work serves a socio-economic purpose and an opportunity to delay marriage [11]. On the employers’ side, with greater numbers of women entering the workforce and a growing middle class in many countries, many households are seeking affordable help by securing the services of domestic workers of all ages [12]. Some households also believe that hosting a child domestic worker is an act of benevolence towards poorer families, especially when girls are sent by their family members. 

The situation of child domestic workers is specifically characterised by an indistinct relationship with the employing family. That is, the girls or adolescents are working, but not considered workers, and although they are living in a family setting, they are not treated like family members. In household surveys, CDW are frequently declared as ‘fostered’ or adopted children doing unpaid household chores [13,14]. This ambiguity creates a care vacuum that generally works against the child’s interests by disguising a familial arrangement that can entail neglect and exploitative working conditions and oftentimes creates and masks various forms of abuse [15].

The precarity of a CDW’s situation is encapsulated in their position as a young, economically disadvantaged, female worker who is largely dependent on the support of employers and others for protection and any potential improvements to her situation. Yet, at the same time [16], studies indicate that being a live-in worker in an unfamiliar urban area can increase girls’ social isolation and separate them from social networks that might, at best, help them flourish and, at least, protect them from harm. For many, the daily experience of discrimination and isolation in the employer’s household is a heavy burden that takes a toll on their mental health [8,17]. 

Studies have highlighted that child domestic workers may suffer a range of psychosocial and physical risk resulting from, inter alia, their long working hours, excessive employer control, and for some, physical and emotional violence [18,19,20,21]. Evidence also indicates that some children in domestic work are extremely vulnerable to street connectedness and sexual exploitation, and some are pushed to these external risks by sexual abuse in the employing households [22,23,24]. Yet, at the same time, studies on child domestic workers from around the world also hint at their wide range of circumstances, from hazardous to beneficial situations [25,26,27]. For instance, findings by Hesketh et al. in their study of child domestic workers in the Philippines and India demonstrate this wide variation in conditions, as 87% of Filipino workers were attending school compared to only 35% in India. Similarly, children’s psychosocial scores also differed considerably, with 36% of Filipino workers scoring in the lowest tercile compared to controls (30%) versus young Indian workers (67% vs. 25%) [27]. 

Reports have also noted the paradox that, while child domestic workers commonly attach great importance to the opportunity to further their education, their daily work demands or employer’s prohibitions often directly impede their chances of formal schooling [28,29,30]. Additionally, a recent scoping study conducted among employers of domestic workers in Myanmar indicated that employers have numerous concerns about permitting girls to participate in activities outside the household. For example, employers suggested that, in addition to losing work time, allowing the girls outside the house puts pressure on the employer’s responsibilities as guardians of their young workers. Employers noted, for instance, that they feared inexperienced rural girls would meet up with undesirable individuals or might become pregnant while under their guardianship [31]. As a result of their highly isolated working situation, it is not uncommon for child domestic workers to have higher drop-out rates, poorer perceptions of their own achievements, an increased likelihood of repeating school years, or potential lifelong illiteracy [32]. 

Despite the development efforts to prevent or eliminate child labour and in spite of the multiple risks to working children’s personal safety and healthy child development, to date, there have been few studies examining interventions to support the education, skills training, and health of young girls who are currently working as domestic workers. Evaluations have been limited for multiple logistical and philosophical reasons, including the considerable investments for rigorous evaluations—funding that often competes with money to produce the interventions. In this vein, there has been a long-standing misunderstanding about the importance of strong evaluations to avoid repetitive ineffective investments. Further, there is often a misguided assumption that evaluations pose inexorable ethical challenges because children must be neglected to create a control group. However, there are numerous methods that overcome these concerns, such as stepped wedge designs, which include segments of the population over time. However, donors must be dedicated to learning about impact, as the budget for true evaluations can be costly in the short term, but beneficial to scale up in the longer term. Philosophically or politically, interventions for working youth may also be viewed as violating the ultimate goal of eradicating child labour. It can be difficult for international agencies promoting conventions and laws on child protection to be seen as facilitating the needs of working youth, rather than solely securing their release. 

The aim of this review was to learn about interventions that have been evaluated and might represent promising methods to access and support girls and adolescents who are working as domestic workers. Specifically, we sought to identify and synthesise peer-reviewed and grey literature on evaluated health, education, and economic interventions that were designed for, or specifically included, child domestic workers, including any interventions specifically targeting employers. 

## 2. Methods

### 2.1. Search Strategy

The protocol was registered in the PROSPERO database of systematic reviews, registration number CRD42019148702 [33]. The protocol search strategy and search terms covered four rapid and scoping systematic reviews on CDWs and employers (the population of interest) and focused on health-, education-, and employer-focused interventions with CDWs, as elaborated elsewhere [34]. A librarian from the London School of Hygiene and Tropical Medicine supported the development of search concepts and terms and searched an overarching set of databases (see Supplementary File S1), which included Web of Science, EMBASE, Global Health, MEDLINE, International Bibliography of the Social Sciences, and Econlit from inception to 4 June 2019. As detailed in Supplementary File S1, two reviewers (C.W.C. and N.S.P.) searched UN agency websites and other grey literature sources for further citations. 

### 2.2. Selection Criteria

At the title and abstract screening stage, as elaborated elsewhere [34], for all four CDW-related reviews, studies were considered eligible if they specifically targeted or included: (1) CDWs (up to 18 years old) and young adult domestic workers (18–25 years old) or employers of CDWs; (2) either occupational outcomes, health, education outcomes, risks or abuses, CDW prevalence, economic outcomes, or outcomes related to employer attitudes or behaviour. Observational studies and intervention evaluations reporting on relevant outcomes for each review were eligible. Studies could include: observational studies (cohort, cross sectional/post only assessments, case-control), experimental studies, or quasi-experimental studies. Systematic reviews were used for forwards/backwards tracking of citations. Studies from low- and middle-income countries were eligible. Though high-income countries, studies from Brunei, Macau, Hong Kong, Taiwan, and Singapore were also included, as migration to these countries from Southeast Asian countries is common. This review contributes to a larger body of work on CDW in Southeast Asia, which is the reason these countries were selected. In order to account for recent evidence, date limits of 1990–2019 were applied. Studies pre-1990 were not relevant. Only studies published in English were eligible. 

Studies were excluded if: (1) their focus was on domestic workers who were aged 25 years or above; (2) addressed children or young adults (up to age 25 years) who were working in their natal homes; (3) did not address any of the relevant outcomes, and (4) did not include an evaluative study design.

### 2.3. Data Extraction

In the initial screen as described in the overarching protocol covering four reviews [34], two reviewers (C.W.C. and N.S.P.) conducted title abstract screening of academic and grey literature citations, labelling studies that were potentially eligible for further screening by the review lead (N.K.). Two reviewers (C.W.C. and N.K.) divided the citations in half, then, screened this refined list of downloaded titles and abstracts for potential inclusion against inclusion criteria in the protocol and screening form as detailed in Supplementary File S2. Each reviewer reviewed the other’s list to assesses potential agreement on ‘maybes’. At the title and abstract screening stage, in order to check the consistency of applying the inclusion criteria, the same two reviewers reviewed the final list of ‘maybes’ again. A random check on 10% of each other’s excluded and included studies was also conducted by both reviewers to ensure consistency. The same two reviewers then used the overarching inclusion criteria to independently assess the potential eligibility of full text papers. Please see Pocock (2021) for more detail [34]. 

C.W.C. developed and piloted a data extraction form and used this to extract data from included papers. Extracted data were then cross-checked by N.S.P for 25% of the studies. Data were extracted on intervention description, inputs, activities, type of evaluation, outcomes, effect size or impact where applicable, limitations, and ethical considerations.

### 2.4. Quality Appraisal

Three reviewers appraised the methodological quality of studies. According to study design, two reviewers, C.W.C. and N.S.P., used the Joanna Briggs Institute (JBI) critical appraisal tools (CAT). The JBI CATs include questions on sampling methods, methods of measuring the outcome, analysis methods, and response rate. Seventy-five per cent of studies were appraised by C.W.C., while N.S.P. appraised the remaining 25% (the same studies for which data were extracted). Fifty per cent of each reviewer’s allocation was then independently appraised by the other to ensure that the quality criteria were being applied consistently. No significant disagreements were found [34]. N.K. also used the Critical Appraisal Skills Programme (CASP) and Cochrane Effective Practice and Organisation of Care (EPOC) appraisal tools according to study design. The CASP includes questions on trial design, randomisation, and statistical analyses and the Cochrane EPOC includes questions on study design, randomisation, statistical analyses, and inferences and blinding. The overall quality of the studies was classified using the following criteria: ‘poor’ 0–50%, ‘moderate’ 51–75%, and ‘good’ 76–100%.

## 3. Results

**Figure 1:**
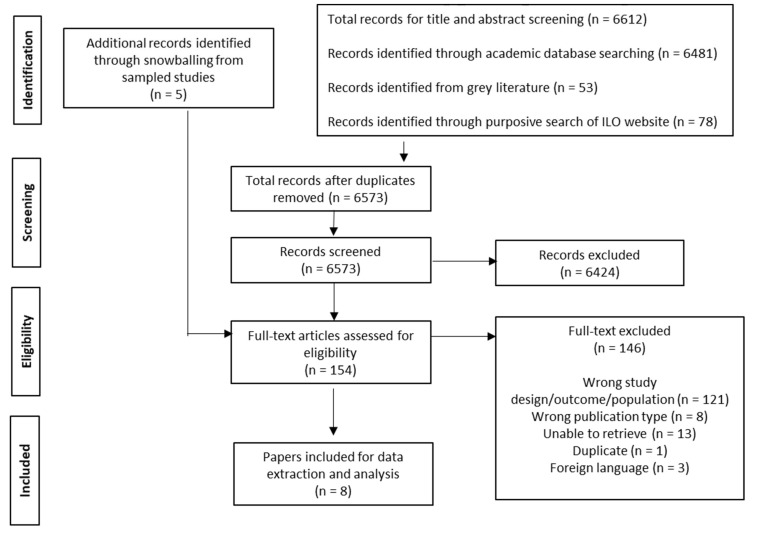
Flowchart of study selection.

The study selection process is summarised in Figure 1. We identified 6612 articles from the academic databases, grey literature, and ILO website searches. Following deduplication, a total of 6573 articles were screened for eligibility. These were divided between three reviewers (C.W.C., N.S.P., and N.K.) who screened the titles and abstracts against inclusion criteria as per our PROSPERO protocol [33]. Of these, 149 progressed to full-text screening. A further five snowballed studies were included, resulting in 154 articles undergoing full-text screening by three reviewers (C.W.C., N.S.P., and N.K.). Eight articles were assessed to meet our inclusion criteria. All included papers were published in English.

Table 1 summarises the key features of the included papers. The eight papers reported on five interventions (Table 1). The five interventions were evaluated using either a randomised controlled trial [35,36,37,38] or a quasi-experimental study design [39,40,41,42]. Interventions included child domestic workers and sought to improve health, education, or economic outcomes.

### 3.1. Health-Related Outcomes

Six papers reported on health-related outcomes. Two studies, Ismayilova et al. (2018) and Karimli et al. (2018) that were assessed to be of moderate and good quality, respectively, reported on evaluations of Trickle Up and Trickle Up Plus [36,37], interventions that were conducted with children aged 10 to 16 from ultra-poor families in Burkina Faso. Children’s involvement in family business or any work for payment was captured by asking the question: ‘During the past week, did you do any of the following activities, even for only 1 hour?’ The measure was coded ‘yes’ if child responded ‘yes’ to any of the following: ‘did you run any kind of big or small business for yourself or with business partners (e.g., selling things, repairing things, hairdressing, barber, shoe-shining, and so forth)’; do any work as a maid for someone who is not a member of your household for any payment?’ [37]. These studies did not provide disaggregated data for child domestic workers and are included here as some of few that report on evaluations that assessed health-related outcomes for working children and specifically include child domestic workers.

The main aim of the Trickle Up intervention was to provide female caregivers with economic strengthening support through the provision of seed capital grants to fund planned livelihood activities. Trickle Up Plus augmented this economic strengthening programme with family coaching, using a curriculum developed by the Burkina Faso Ministry of Social Action and delivered over five months to address topics including schooling, violence and abuse, trafficking, worst forms of child labour, forced/early marriage, and begging. The evaluation of Trickle Up found no significant effect on children’s work-related health outcomes or work-related abuse at 12 or 24 months. Furthermore, there was no significant effect of Trickle Up Plus on work-related health outcomes at 12 or 24 months. However, for depression symptoms, the findings suggest that compared to the control group and those enrolled on Trickle Up, children exposed to the Trickle Up Plus intervention showed a significantly greater reduction in depressive symptoms at 12 and 24 months (Trickle Up Plus vs. control; 12 months: Cohen’s *d* –0.41, *p* = 0.001, 24 months *d* –0.39, *p* = 0.025; Trickle Up Plus vs. Trickle Up: 12 months *d* –0.22, *p* = 0.020). Trauma symptoms also significantly reduced in the Trickle Up Plus arm compared to the control group (Trickle Up Plus vs. control: 12 months IRR (incidence risk ratio) 0.62 [0.41, 0.92], *p* = 0.018). Additionally, the findings suggest that while there was no effect on work-related abuse at 12 months after the intervention, there was evidence of a slight reduction at 24 months (verbal abuse only) (Trickle Up: At 12 months OR 1.0 [0.4 to 2.5]; at 24 months OR 1.2 [0.4, 3.3]. Trickle Up Plus: at 12 months OR 0.8 [0.3, 2.2]; at 24 months OR 0.1 [0.03, 0.7], *p* < 0.05). 

Two other papers, by Erulkar et al. (2013) and Erulkar and Medhin (2014), that were assessed to be of moderate quality, reported on evaluations of Biruh Tesfa and Powering Up Biruh Tesfa [41,42], which targeted vulnerable girls and adolescents aged between 10 and 19 in Ethiopia. It is important to note that neither of these studies reported disaggregated data for child domestic workers, although a significant proportion (approximately 50%) of the study participants were identified as domestic workers. The aim of the Biruh Tesfa intervention was to establish social support networks and provide life skills training for vulnerable out-of-school girls. The main activities were the training of female mentors who subsequently conducted house-to-house recruitment of participants into girls’ ‘safe spaces’ groups. In these groups, participants were supported to cover topics including life skills training, self-esteem, financial and health literacy, hygiene and menstruation, and disabilities and violence. They were also able to access a voucher scheme for free medical consultations and medications and access essential items such as sanitary items, basic clothing, and stationery. The Powering Up Biruh Tesfa was an expansion of the Biruh Tesfa programme into 20 new districts within five sub-cities of Addis Ababa and included a particular focus on literacy and numeracy skills and health service utilisation. The evaluations of these interventions suggested that participation in Biruh Tesfa and Powering Up Biruh Tesfa was associated with greater improvement in HIV knowledge and knowledge about where to access voluntary counselling and testing (VCT) services at endline in the intervention arm compared to the control arm (HIV knowledge at endline, intervention vs. control: OR 1.93 [1.23, 3.02], *p* < 0.01. Knowledge of where to obtain VCT at endline, intervention vs. control: OR 2.03 [1.31, 3.13], *p* < 0.01). Being in the intervention group was also associated with higher odds of health services utilisation as compared to the control group (adjusted odds of health service utilisation in the past six months, intervention vs. control: OR 1.55 [1.22, 1.98], *p* < 0.001). 

Finally, two papers by Engrebretson (2012) and Engrebretson (2013), that were evaluated to be of poor quality, reported on Filles Eveillees (Girls Awakened) [39,40], described the results of an intervention with female adolescent migrant domestic workers aged 11–18 in Burkina Faso. The aim of Filles Eveillees was to provide participants with a safe space to access peers and female mentors in order to increase their economic opportunities and reduce their vulnerabilities. Specifically, the intervention sought to increase participants’ social capital and build their skills in health (including sexual and reproductive health) life skills, financial capabilities, and to link girls to services. At endline, positive changes were reported in social capital (as measured by the questions ‘has a safe place to meet friends’ ‘has someone to borrow money from in an emergency’ and ‘has people to talk to for advice’) and self-confidence (97% agreed they had more female friends; 95% had more confidence in expressing self), as well as attitudes towards health seeking and HIV screening (97% agreed that health facilities were girl-friendly (vs. 86% baseline); 92% had favourable attitudes towards HIV testing (vs. 73% baseline)). Improvements were also observed in HIV and sexual health knowledge and knowledge about HIV services in the community (42% could name two correct preventative methods vs. 16% at baseline)). This intervention did not have a control group.

### 3.2. Education-Related Outcomes

Two papers, by Erulkar (2013) and Erulkar and Medhin (2014) [41,42], reported on education-related outcomes. Powering Up Biruh Tesfa in Ethiopia reported significant improvements in literacy and numeracy scores from baseline to endline in the intervention group, amongst those who had never attended formal schooling (intervention group *n* = 127, control group *n* = 74), and an improvement in mean literacy/numeracy score (range 0 to 1): adjusted mean difference (adjusted for age, migration status, and relationship to household head) was 0.08 [0.02, 0.14], *p* < 0.05). A third paper, by Stark et al. (2018) was judged to be of moderate quality and reported on findings related to the evaluation of the Creating Opportunities through Mentoring, Parental Involvement, and Safe Spaces (COMPASS) [38] intervention in Ethiopia. COMPASS is a social empowerment programme developed by the International Rescue Committee and sought to build participants’ (predominantly social) assets with the aim to reduce their economic vulnerability, prevent violence, and to promote learning and peer interaction in safe spaces. The main intervention components included weekly mentor-facilitated group meetings and engagement with the caregivers of COMPASS beneficiaries through monthly discussion groups. COMPASS involved vulnerable adolescent refugees aged between 13 and 19 from surrounding conflict-affected regions, mainly Sudan and South Sudan. This intervention did not detect any significant intervention effects on children’s school enrolment during the most recent school year (intervention vs. control: adj OR 1.21 [0.82, 1.82]) and did not provide disaggregated data for child domestic workers. 

### 3.3. Economic Outcomes

Three papers, by Stark (2018), Engrebretson (2012), and Engrebretson (2013) [38,39,40], reported on economic outcomes. The evaluation of COMPASS reported on the effect of an economic strengthening intervention on the economic vulnerability of adolescent refugee girls in Ethiopia aged 13 to 19, which included paid child domestic workers. The study assessed four primary indicators: (1) school enrolment during most recent school year; (2) working for pay in the past 12 months; (3) experiencing transactional sexual exploitation over the past 12 months; (4) an aggregate of the first two [38]. The findings suggest that at endline, the odds of being enrolled in school or working for pay were higher, and the odds of engaging in transactional sexual exploitation were lower for those who were enrolled on the programme compared to the control group. However, these findings were not statistically significant (working for pay at endline, intervention vs. control: adj OR 1.04 [0.73, 1.48]; transactional sex, endline: intervention vs. control: adj OR 0.62 [0.30, 1.26]). Finally, the evaluations of the Filles Eveillees intervention reported some improvements in financial behaviour, namely, saving behaviour, between baseline and endline (74% vs. 84%, *p* < 0.05).

### 3.4. Employer-Related Interventions

No evaluations of employer-related interventions were identified to be included in this review.

## 4. Discussion

The first and most evident finding from this review is that there have been extraordinarily few evaluations of interventions designed for youth who are currently working as domestic workers for a number of resources and logistical, ethical, and political reasons. While each intervention identified by this review included child domestic workers, only one, Filles Eveillees, was specifically targeted at child domestic workers. By including youth in a variety of risk situations, we are limited in what intervention knowledge we can glean about how to reach young domestic workers because they are likely to have different challenges to intervention uptake and participation than other young people. For example, as discussed, child domestic workers’ time and availability are generally under the control of their employers, so most interventions will have to include a component to negotiate with the employer. Further, there are likely to be differences in health and social support needs between girls who are living with their own parents versus girls who have migrated away from home.

A further disappointing finding from this review is that even when domestic workers were included in interventions, the data were not disaggregated to understand the particular effects the activities had on child domestic workers. This would have been particularly valuable because circumstances for children working in different sectors will influence the types of intervention approaches that can reach them and from which they can benefit. Interventions will have to be customised to meet their particular circumstances. Moreover, child domestic workers in particular are often a more ‘hidden’ and less accessible group than youth working in other sectors, and their access to interventions will often have to be negotiated with their employers, not least because they live on the premises and are often required to work full days or unpredictable hours. Interventions will have to be designed to meet these particular circumstances and challenges.

These gaps are not surprising, given the poor measurement of child domestic work globally [34], as suggested by a recent review indicating that child domestic workers are likely to be severely undercounted in most household surveys, for reasons including reliance on household heads to accurately describe the occupation and industry of household members, rather than asking specific questions on tasks and payment [34]. When CDWs are undercounted, policymakers and donors may be less inclined to invest in policies or interventions targeting them relative to more visible forms of children’s work.

If data on child domestic workers had been analysed in all intervention studies included in this review, findings might have shown that the activities had different effects on their intervention uptake, participation in education, or mental health features, depending on their employer-required time restrictions or work conditions. Other research has shown that opportunities for domestic workers to participate in education programs seem to depend on the context. For example, a study by Hesketh et al. regarding child domestic worker health in India and the Philippines indicated that 87% of workers in the Philippines were attending school compared with 35% of those in India [27]. 

On the other hand, several studies that were ranked higher in quality showed some promising effects of interventions among girls who are especially vulnerable to poor health, harm, and exclusion. For example, the evaluation of the Trickle Up programs in Burkina Faso demonstrated reductions in depression and trauma symptoms. Additionally, the Biruh Tesfa intervention in Ethiopia fostered greater HIV knowledge and better health service utilisation. Moreover, Biruh Tesfa was also able to show an increase in girls’ literacy and numeracy. In literature on child domestic work, loss of education opportunities is often cited as a critical problem associated with child domestic work [43], so improvements in these basic educational aspects are an important aspect of any intervention. Additionally, two studies showed financial improvements for intervention participants, which is especially meaningful, as previous research with human trafficking survivors indicated that lost or cheated income was predictive of poor mental health outcomes [44]. 

Importantly, we were unable to identify evaluations of interventions targeted specifically at employers. However, we are aware of an assessment of the influence of district bylaws on employer behaviour in Tanzania as well as work targeting employers directly in Bangladesh both of which appear to have had some positive outcomes [45,46]. To date, there is scant evidence on employer-focused interventions, which makes it difficult to know how these types of interventions might operate independently or in tandem with youth-focused activities. At present, it seems that there is very little research to inform interventions, for example, by learning how employers view the educational opportunities for young workers or options for enhancing their future livelihoods. Indeed, Klocker (2014) notes how employers’ perspectives remain side-lined in research on child domestic work [47]. Moreover, we found no interventions addressing labour intermediaries or brokers who help place youth with employers. 

Finally, at present, we could not identify evidence about costs or cost effectiveness of any interventions for children working as domestic workers. We believe these types of data will be crucial to future investments in various intervention designs. 

### Limitations

This review has a number of limitations including the fact for some of the included studies, and we do not have disaggregated data for child domestic workers, which reflects two outstanding challenges: (1) that few interventions are specifically targeted at CDWs, for whom specific and negotiated outreach is required with employers and (2) a paucity of evidence on interventions that have been evaluated that might represent promising methods to access and support girls and adolescents who are working as domestic workers. We have also only included interventions for which there was a published evaluation. We are aware that there are many interventions that have focused on or included child domestic workers, but which have not been evaluated yet. For this review, it was important that we could identify the effectiveness of any interventions versus simply describing various designs. It is possible, perhaps even likely, that there are effective interventions for which an evaluation would be invaluable. It is also worth noting that we did not examine the various pre-departure interventions that seek to prevent children’s entry into various forms of labour, including domestic work. We believe these types of intervention will have very different challenges, should be embedded into development and poverty reduction programming, and are unlikely to help the millions of youth who are already working as domestic workers. 

## 5. Conclusions

Overall, the findings of this review highlight that there are few evaluated interventions to improve health and education outcomes for children who are currently working as domestic workers. Given that there appear to be over 17.2 million children currently working as domestic workers or as termed in other locations as *petites bonnes* or *confiage* (west Africa), *vidomegon* (Benin), and *restaveks* (Haiti), it seems more than time to learn the best ways to reach these young women and girls to help them prepare for a more promising future. Certainly, there is a need for a strong policy or legislative foundation. However, these policies must be accompanied by interventions, particularly government-supported programs, that have the capacity to provide education, vocational skills training, and other forms of support to either help girls return to full-time education or to facilitate education and skills for better jobs among girls who are already engaged in domestic work. Research that poses the broad questions such as ‘what works to reduce child domestic work’ must be accompanied by studies that pose questions such as: What are safe and effective ways to reach children in domestic work? How can interventions negotiate access with employers? What skills or knowledge do young domestic workers want to learn and how do they want to learn it? What types of jobs in the local market will provide incomes for a sustainable livelihood and what training is needed to enter these job markets?

It is relatively clear to all who work on child domestic work that measures to immediately eradicate child domestic work are not realistic or feasible—not least because it might mean that girls move into situations that are more hazardous. Moreover, studies from around the world indicate that while some youth are in extraordinarily abusive conditions, others may be in situations that have multiple benefits compared to their prior living conditions at their place of origin [26,27,48]. These potentially positive circumstances are what make it so important to identify ways to reach those youth who can benefit from programs that foster skills and knowledge that can improve their future livelihoods and independence. Thus, until the time when families and girls have better opportunities to remain in education or find safe and lucrative work, employment regulations combined with appropriate resources for alternative education are needed to improve the current conditions imposed by employers, especially limited working hours so girls can participate in education or skills building *and* have leisure time, fair wages, and decent treatment to protect their physical and mental health. Importantly, complex intervention approaches of this kind also need to be accompanied by strong intervention-focused research and evaluation to understand what works to safeguard the health and livelihood outcomes for child domestic workers, especially to help girls and young women make choices about their future.

## Figures and Tables

**Table 1:** Peer-reviewed papers on child domestic worker interventions (*n* = 8).

Author (Year)	Intervention	Country	Study Design	Study Population	Sampling Method	Outcome	Disaggregated Outcomes for CDWs	Study Quality
Karimli et al. (2018) ^a^ [37]	Trickle Up and Trickle Up Plus	Burkina Faso	Open-label cluster randomised design	Ultra-poor families (mothers and children) children aged 10–15	Random selection	Health and social	No	Moderate
Ismayilova et al. (2018) ^a^ [36]	Trickle Up and Trickle Up Plus	Burkina Faso	Open-label cluster randomised design	Ultra-poor families (mothers and children) children aged 10–15	Random selection	Health and social	No	Good
Erulkar (2013) ^b^ [41]	Biruh Tesfa	Ethiopia	Quasi-experimental (pre and post intervention in control and intervention areas)	Vulnerable girls and adolescents aged 10–19	Purposive	Health and social	No	Moderate
Erulkar and Medhin (2014) ^b^ [42]	Powering Up Biruh Tesfa	Ethiopia	Quasi-experimental (pre and post intervention in control and intervention areas)	Vulnerable girls and adolescents aged 10–19	Random selection from eligible girls	Educational	No	Moderate
Engrebretson (2012) ^c^ [39]	Filles Eveillees	Burkina Faso	Pre–post evaluation (no control)	Female adolescent migrant domestic workers aged 11–16	Purposive	Health and social	Yes	Poor
Engrebretson (2013) ^c^ [40]	Filles Eveillees	Burkina Faso	Pre–post evaluation (no control)	Female adolescent migrant domestic workers aged 11–18	Purposive	Health and social	Yes	Poor
Covarrubius (2012) [35]	Malawi Social Cash Transfer Programme	Malawi	Open-label cluster randomised design	Children from impoverished households eligible for participation in Malawi’s social cash transfer programme aged <18	Villages randomly assigned, households assigned using criterion sampling	Prevalence	Yes	Poor
Stark (2018) [38]	COMPASS (Creating Opportunities through Mentoring, Parental Involvement, and Safe Spaces)	Ethiopia	Randomised controlled trial	Vulnerable adolescent refugees from surrounding conflict-affected regions mainly in Sudan and South Sudan aged 13–19	Random assignment	Economic	No	Moderate

^a^ Same study; ^b^ Same study; ^c^ Same study.

## Data Availability

Data are contained within the article and Supplementary Materials.

## Supplementary Materials

The following are available online at https://www.mdpi.com/article/10.3390/ijerph181910084/s1, Supplementary File S1: Searches, Supplementary File S2: Screening Protocol.
