# Supplementary material for: Evaluations of Interventions with Child Domestic Workers: A Rapid Systematic Review
Source: Int J Environ Res Public Health. 2021 Sep 25;18(19):10084. doi: 10.3390/ijerph181910084 (PMC8508353; doi:10.3390/ijerph181910084)
Supplement: Supplementary file 1 [file ijerph-18-10084-s001.zip › Supplementary File 2_Screening Protocol.pdf]

Supplementary File S2. CDW Reviews Screening Protocol

1. Title & Abstract screening

Instructions (CC & NP):

Proceed through the questions in order. Note that an "unclear" answer never excludes a study. The questions are designed to be as objective as possible. The questions are meant to start with those easier to ascertain and progress to those that will be harder to answer based on a quick read. The screener should feel confident of any "yes" or "no" answer used to exclude a study.

If you cannot conclusively say "yes" or "no", please mark the study as unclear and it will move on to the next level of screening. You may decide to mark the study with one of the "Maybe" labels for each review.

***Eligible HICs: Singapore, Taiwan, Macau, Hong Kong, Brunei. LMICs according to World**

Screening questions – Title & Abstract	No	Yes	Unclear
1. Does the abstract or title mention CDWs, Domestic Workers, or their employers?			
IF NO, THEN EXCLUDE			
2. Does the study focus on an LMIC, or one of the eligible HICs?*			
IF NO, THEN EXCLUDE			
3. Is the study an eligible study design?**			
IF NO, THEN EXCLUDE			
4. Is the study a Systematic Review?			
IF YES, INCLUDE & LABEL > systematic review			
5. Does the study mention a health, education intervention with CDWs or DWs, or their employers?			
IF YES, INCLUDE & LABEL > 2: interventions			
6. Does the study mention CDW pathways and any outcomes (occupational, social, financial) AFTER CDW?			
IF YES, INCLUDE & LABEL > 1: trajectories			
7. Does the study mention a quantitative survey or measurement of CDW prevalence, health outcomes, risks, abuses?			
IF YES, INCLUDE & LABEL > 3: measurement tools			
8. Does the study try to quantify macroeconomic indicators of CDW or DW at the country or sub-national level?			
IF YES, INCLUDE & LABEL > 4: economics			

Bank

****Eligible study designs: observational (cohort, case-control, cross sectional), qualitative studies, quasi experimental and experimental. Intervention evaluations using any of these designs are eligible. Systematic reviews. Macroeconomic studies, either conceptual or empirical using household data.**

Labels:
1: trajectories
2: interventions
3: measurement tools

4: economics
systematic review

Maybe 1: trajectories

- Includes qualitative studies on CDWs generally
- Includes studies with CDW/DW returnees generally

Maybe 2: interventions

- Includes descriptive studies on employer, recruiter or public attitudes towards CDW or DW
- Includes media analyses of depictions of CDW, DW

Maybe 3: measurement tools
Maybe 4: economics

- Includes studies on remittances where they mention CDW or DWs overseas
- Includes studies with large surveys of CDW/FW

policy
street kids
child marriage

- Studies mentioning CDW as alternative pathway to CM

For age, please include all studies for now if they mention Domestic Workers (DW). Studies may include domestic workers aged 25 or below. Filtering for this comes at full text stage – where if only adults aged 25 or over are included, then EXCLUDE the study.
Review leads may opt to refine inclusion/exclusion criteria at full-text screening stage, according to their preference.
